# Changes in Degree Centrality and Functional Connectivity after the First Cycle of Neoadjuvant Chemotherapy in Newly Diagnosed Breast Cancer: A Longitudinal Study

**DOI:** 10.1155/2022/8270100

**Published:** 2022-11-28

**Authors:** Yixin Hu, Daihong Liu, Yongchun Deng, Feng Yu, Hong Yu, Yong Tan, Xiaoyu Zhou, Jiang Liu, Weiwei Lei, Xiaohua Zeng, Jiuquan Zhang

**Affiliations:** ^1^Department of Radiology, Chongqing University Cancer Hospital & Chongqing Cancer Institute & Chongqing Cancer Hospital, Chongqing 400030, China; ^2^Department of Breast Cancer Center, Chongqing University Cancer Hospital, Chongqing, China; ^3^Chongqing Key Laboratory for Intelligent Oncology in Breast Cancer (iCQBC), Chongqing University Cancer Hospital, Chongqing, China; ^4^Department of Intensive Care, Chongqing University Cancer Hospital & Chongqing Cancer Institute & Chongqing Cancer Hospital, Chongqing 400030, China

## Abstract

**Purpose:**

To evaluate the longitudinal changes of brain degree centrality (DC) and functional connectivity (FC) in breast cancer patients after the first cycle of neoadjuvant chemotherapy (NAC).

**Methods:**

Thirty-five breast cancer patients were included in the NAC group. Resting-state functional magnetic resonance imaging (rs-fMRI) and neuropsychological test were performed at baseline before NAC (time point 0, TP0) and after the first cycle of NAC (time point 1, TP1). The healthy controls (HC) included 30 healthy subjects and received the same rs-fMRI scan and neuropsychological test as the above-mentioned NAC group at one time point. DC and FC analyses were conducted to assess brain connectivity of all participants. Receiver operating characteristic (ROC) curve was used to assess the ability of DC and FC in distinguishing patients before and after chemotherapy.

**Results:**

In the NAC group, the Self-Rating Anxiety Scale scores decreased significantly over time. At TP0 and TP1, the Digital Span Test forward score of the NAC group was significantly lower than that of the HC group. In the NAC group, DC in the right middle frontal gyrus and left precentral gyrus/middle frontal gyrus decreased significantly at TP1, and FC between the left precentral gyrus/middle frontal gyrus and bilateral precuneus was significantly reduced at TP1. Through ROC analysis, we found that the area under the curve (AUC) of DC, FC, and the combined model in distinguishing patients in TP0 or TP1 was 0.7886, 0.7665, and 0.8278, respectively.

**Conclusions:**

Brain connectivity, involving executive and motor function related brain areas, changes in the short term after NAC treatment in breast cancer patients.

## 1. Introduction

Breast cancer is the most prevalent cancer worldwide according to the International Agency for Research on Cancer GLOBOCAN cancer statistics for 2020 [1]. As an essential and effective treatment, chemotherapy is associated with cognitive impairment in breast cancer patients, with 8.1%-75% of survivors suffering from this side effect [2]. Cognitive problems present major challenges to patient care and quality of life. Elucidating the underlying neurological mechanisms of chemotherapy-related cognitive impairment may enable early diagnosis and treatment.

Comprehensive cognitive domains are involved in chemotherapy-induced cognitive impairments, including attention, memory, executive function, and information processing speed [3]. The potential mechanisms underlying the cognitive deficits have been explored in previous studies. For instance, postchemotherapy patients exhibit high levels of proinflammatory cytokines and oxidative stress, which have been suggested to be associated with cognitive deficits [4]. In addition, the neurotoxicity of chemotherapeutic agents can cause neurogenesis inhibition and impaired white matter integrity [5]. Other potential factors should also be taken into consideration, including DNA damage, mitochondrial dysfunction, and aberrant neurotransmitter levels [5, 6]. These interrelated factors may coordinately contribute to the development of cognitive impairment. However, the mechanism is only beginning to be explored. Neuroimaging is also an essential approach for the exploration of chemotherapy-associated large-scale neuronal abnormalities that may underlie cognitive impairment in breast cancer patients.

Functional magnetic resonance imaging has been proven to be an informative neuroimaging method to determine brain function alterations in patients receiving chemotherapy. Systemic chemotherapy for breast cancer is inferred to comprehensively influence the brain. Among the neuroimaging measurements, degree centrality (DC) is a measurement method based on graph theory, which quantified the number of links connected to a node and used to assess the importance of each node in brain network, in terms of its connectivity number to every voxel [7], this underlies the importance of the local region, termed the “brain hub” [8]. It reduces the possible bias caused by selecting brain regions according to the priori assumption and exhibits relatively high test-retest reliability [9]. Recently, the DC approach has been successfully used to disclose the neurobiological mechanism for several diseases, such as obsessive compulsive disorder [10], Alzheimer's disease [11], and major depressive disorder [12]. Breast cancer survivors have been reported to have reduced DC of gray matter networks in the frontotemporal regions, which may be associated with impairments of memory and executive functioning [13]. Furthermore, FC analysis enables an exploration of the particular edges that showed aberrant connections to these nodes, as we demonstrated previously [8]. A recent study combined DC and FC to derive that the dorsolateral prefrontal cortex may play a key role in multiple system atrophy with cognitive impairment [14]. These results indicate that DC and FC are feasible methods to explore the damage of the whole brain neural network in the resting state. However, as far as we know, there is a lack of relevant studies using the above methods to explore the damage of the whole brain neural network in breast cancer patients. The characteristics of DC and FC anomalies caused by NAC remain to be further studied.

Previous neuropsychological studies suggest that cancer patients have cognitive dysfunction at all stages of the disease course, and subjective complaints are most frequently reported one month after the end of chemotherapy [15], and the time point of most neuroimaging studies is also one month after the end of chemotherapy [16, 17]. A recent study showed a sharp decline in cognitive function of breast cancer patients within three months after chemotherapy [18]. However, the short-term changes in brain connectivity caused by NAC remain to be studied. In this study, breast cancer patients completing the first circle of NAC were enrolled to investigate the short-term effect of chemotherapy on the brain and exclude the potential influence of surgery. We hypothesized that the aberrant brain connectivity may contribute to the development of cognitive impairment in breast patients receiving chemotherapy. We used the regions that showed significant alterations in DC, combined with secondary seed-based FC analysis to explore the changes of brain connectivity after the first circle of NAC in breast cancer patients. Finally, the receiver operating characteristic (ROC) curve analysis based on logistic regression was constructed to assess the ability of DC values and FC *z* scores to distinguish patients between before and after chemotherapy.

## 2. Materials and Methods

### 2.1. Participants

This prospective longitudinal study was approved by the Ethics Committee of Chongqing University Cancer Hospital (approval number: czls2021042-A). All participants provided written informed consent. Thirty-five female breast cancer patients (NAC group) were recruited from our institution, and thirty female healthy controls (HC group) were recruited from communities between January 2021 and June 2021. The inclusion criteria of the NAC group in this study were as follows: (1) stage I-III breast cancer patients between the ages of 20 and 70 and scheduled for NAC before surgery and (2) right-handedness. Exclusion criteria were as follows: (1) organic brain abnormalities (brain metastases, trauma, stroke, or a history of brain surgery); (2) history of neurological or psychiatric diseases (major depressive disorder, dementia, multiple sclerosis, schizophrenia, and epilepsy); (3) metal implants; and (4) contraindication of MR examination. We recruited HC groups matched by age, gender, and education level. None of them had a history of cancer or chemotherapy, and the remaining exclusion criteria were the same as those in the NAC group.

In the NAC group, the baseline assessment was performed before NAC (time point 0, TP0), including a brain rs-fMRI scan and neuropsychological test. Follow-up evaluation was performed after the first cycle of NAC (time point 1, TP1). The HC group underwent the same brain rs-fMRI scan and neuropsychological test at one time point. Demographic information of the study participants, including age, education, body mass index (BMI), blood pressure, and menstrual status, was obtained through a self-report questionnaire. In addition, we obtained disease stage and treatment information, such as the chemotherapy regimen, from the NAC group through medical record abstraction.

### 2.2. Neuropsychological Test

All participants underwent a battery of neuropsychological test in a fixed order that assessed their global cognitive level and major cognitive subdomains. The major cognitive subdomains were evaluated with the following test: (1) the Trail Making Test (TMT, Part A) for executive function and psychomotor speed [19], (2) the Verbal Fluency Test (VFT) for mental flexibility [20], and (3) the Digit Span Test (DST, forwards and backwards) for working memory [20]. We used the Functional Assessment of Cancer Therapy Cognitive Function Version 3 (FACT-Cog) (version 3, simplified Chinese) [21] to evaluate self-perceived cognitive function. FACT-Cog is designed for cancer patients 18 years and older with chemotherapy-induced cognitive problems. It assesses cognitive complaints over the last seven days. The higher the score, the lower the cognitive complaints. The FACT-Cog includes four subscales: perceived cognitive impairment (PCI), comments from others (Oth), perceived cognitive abilities (PCA), and impact on quality of life (QOL). The Self-Rating Anxiety Scale (SAS) and Self-Rating Depression Scale (SDS) were used to assess anxiety and depression in the emotional test.

### 2.3. Magnetic Resonance Imaging

MRI sequences were acquired with a 3.0 T scanner (Magnetom Prisma; Siemens Health care, Erlangen, Germany) equipped with a 64-channel head-neck coil. Subjects were instructed to close their eyes, stay awake, and avoid thinking about any topics. Earplugs and cushions were used to alleviate noise influence and restrict head motion, respectively. The rs-fMRI data were acquired using rapid gradient echo-planar pulse imaging with the following parameters: repetition time = 2000 milliseconds (ms), echo time = 30 ms, flip angle = 70°, field of view = 240 × 240 mm^2^, slices = 36 (interleaved), matrix = 80 × 80, and voxel size = 3 × 3 × 3 mm^3^. From each participant, 240 volumes were acquired over 8 minutes and 8 seconds. High-resolution T1-weighted structural images were scanned by three-dimensional magnetization prepared rapid gradient echo, and the parameters were as follows: repetition time = 2100 ms, echo time = 2.26 ms, flip angle = 8°, field of view = 256 × 256 mm^2^, slices = 192, matrix = 256 × 256, and voxel size = 1 × 1 × 1 mm^3^. The total scanning time was 4 minutes and 53 seconds.

### 2.4. Neuroimaging Processing

Rs-fMRI data were preprocessed using Data Processing & Analysis for Brain Imaging (DPABI_V5.1) based on MATLAB R2016a [22]. The preprocessing process is as follows: (1) all DICOM files were converted to NifTI files. (2) The first 10 time points of the functional image were removed to ensure high homogeneity of the remaining image. (3) Slice timing was performed to correct the time differences between slices. (4) Realignment was performed to correct head motion, and a report of head motion was created. Any subjects with head motion > 3.0 mm in any direction of *x*, *y*, and *x* or >3.0° at any angle were excluded from the subsequent statistical analyses. (5) The Friston 24-parameter model was applied to regress out head motion effects. Other nuisance variables, including white matter signal and cerebrospinal fluid signal, were regressed out. Individual functional images were normalized into the Montreal Neurological Institute (MNI) space for intersubject comparison [23]. (6) The data were bandpass filtered (0.01–0.10 Hz) to reduce physiological noise at other frequency bands.

Based on preprocessing, DC calculations were performed using DPARSF in a voxel-wise manner with a threshold of *r* > 0.25 in accordance with previous studies [24, 25]. The weighted DC of each voxel was divided by the global mean weighted DC of each subject to achieve standardization. After calculation, all the resulting DC maps were spatially smoothed (full-width at half maximum = 6 mm). According to the follow-up statistical results, brain regions with significantly altered DC were used as seed regions including the brain regions with significant differences in DC over time in the NAC group and the brain regions with significant differences in DC between the NAC group and the HC group. Then, gray matter volume from seed regions was extracted. On the basis of preprocessed data with smoothing (full-width at half maximum = 6 mm), the time series correlation coefficient between seed region and other brain voxels was calculated, and the FC pattern of each subject was obtained. Fisher's *r*-to-*z* transformation was performed to improve the normality of the correlation coefficient values of FC.

### 2.5. Statistical Analysis

SPSS software (22.0 version, IBM Corp., Armonk, NY) was used to analyze the numerical data, including demographic data and neuropsychological test scores. First, the Kolmogorov-Smirnov test was used to check whether the data conformed to a normal distribution. Then, according to the results of the test, comparison analyses were carried out. For the intergroup comparison of the NAC group and HC group (TP0 vs. HC, TP1 vs. HC), an independent sample *t*-test was used for the data conforming to the normal distribution, and the Mann–Whitney *U* test was used for the abnormal distribution. For the intragroup comparison (TP0 vs. TP1) of the NAC group, a paired sample *t*-test was used to analyze the data conforming to a normal distribution, and the Wilcoxon signed-rank test was used to analyze the data with a nonnormal distribution.

Analyses of DC and FC maps were conducted in the Statistical Analysis module of DPABI V5.1 software. First, one-sample *t*-test was performed to check the distribution patterns of DC and FC of each group (compared to “0”). For the intergroup comparison of the NAC group and HC group (TP0 vs. HC, TP1 vs. HC), an independent sample *t*-test was used to compare the DC and FC maps. For the intragroup comparison (TP0 vs. T1) of the NAC group, a paired sample *t*-test was used to compare the distribution of DC and FC. Age, years of education, and head motion parameters were included as covariates. The results were corrected for multiple comparisons using Gaussian random-field theory (voxel level *P* < 0.001 and cluster level *P* < 0.05, two tailed). The gray matter volume of seed regions was compared by paired sample *t* test using SPSS software. The mean DC values and FC *z* scores of significant altered brain regions in NAC group were extracted, and the receiver operating characteristic (ROC) curve analysis based on logistic regression was applied to identify different imaging characteristics of the corresponding groups. Diagnostic accuracy was indicated by the area under the curve (AUC), with values between 0.5 and 0.7, 0.7 and 0.9, and >0.9 having low, moderate, and high accuracy, respectively.

We calculated the average DC values and FC *z* scores of aberrant brain regions and used Spearman correlation analysis to evaluate the correlation between these two parameters and neuropsychological test scores with significant differences. *P* < 0.05 was considered statistically.

## 3. Results

### 3.1. Demographic Characteristics and Clinical Data

Thirty-eight breast cancer patients were recruited in this study. Three of the patients were excluded, one patient had head movement greater than 3 mm during baseline scanning, one patient had head movement greater than 3 mm in the follow-up scanning, and another patient had a history of schizophrenia. Therefore, 35 patients were finally enrolled for data analysis. Among them, three patients refused neuropsychological test at TP0, and three patients refused neuropsychological test at TP1, but they agreed to conduct rs-fMRI data collection and analysis. A total of 29 patients underwent neuropsychological test at both TP0 and TP1. The median time interval between the TP0 and TP1 moments of the MRI scan was 27 days (range 19–46 days). In addition, age, education, BMI, blood pressure, and menstrual status of the HC group were not significantly different from those of the NAC group (*P* > 0.05). The clinical stages and preoperative chemotherapy regimens in the NAC group are detailed in Table 1.

### 3.2. Neuropsychological Test Comparison

The DST forward scores of the NAC group at two time points were significantly lower than those of the HC group (Tables 2 and 3). In the NAC group, the SAS scores decreased significantly over time (*P* < 0.05) (Table 4).

### 3.3. DC and FC Analysis

**Figure 1:**
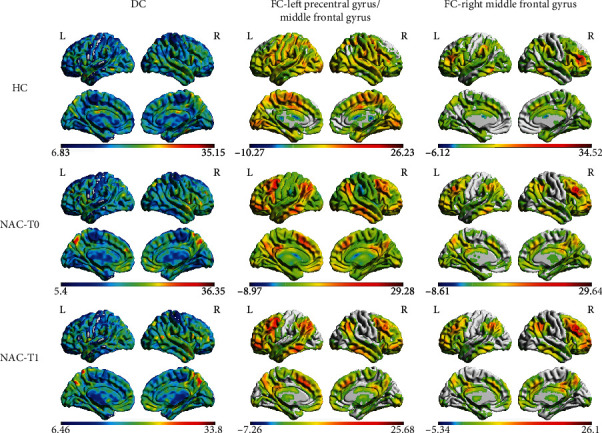
Degree centrality (DC) and functional connectivity (FC) distribution in the HC group and NAC group (one-sample *t*-test). The DC and FC spatial distributions in the NAC group (both time points) were similar to those of the HC group. *P* < 0.05 (corrected with Gaussian random-field theory). The color bar denotes the *t*-value. R: right; L: left.

After correcting the Gaussian random field, the distribution patterns of DC and FC for each group are shown in Figure 1 according to the results of the one-sample *t*-test. It was found that high DC values mainly occurred in the bilateral prefrontal cortex, the precentral gyrus, the postcentral gyrus, and parietal cortex. The left precentral gyrus/middle frontal gyrus mainly connected to the bilateral prefrontal cortex and the temporal lobe and parietal cortex. The right middle frontal gyrus mainly connected to the prefrontal cortex, the parietal cortex, and the temporal lobe. The spatial distributions of DC and FC in the NAC group (both time points) were similar to those of the HC group.

**Figure 2:**
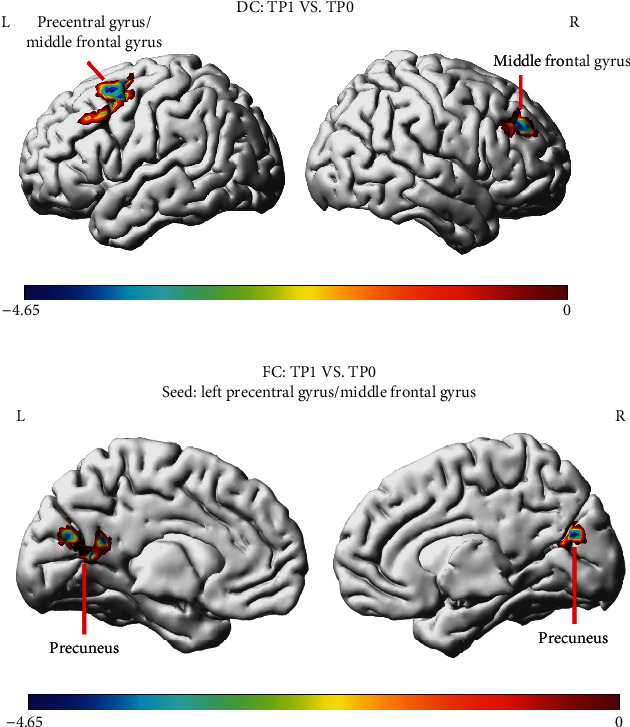
Comparisons of DC and FC distribution between TP0 and TP1 in the NAC group (paired sample *t*-test). (a) The DC in the right middle frontal gyrus and left precentral gyrus/middle frontal gyrus decreased significantly from TP0 to TP1. (b) The FC between the left precentral gyrus/middle frontal gyrus and bilateral precuneus was significantly reduced from TP0 to TP1. *P* < 0.05 (corrected with Gaussian random-field theory). The color bar denotes the *t*-value. R: right; L: left.

**Figure 3:**
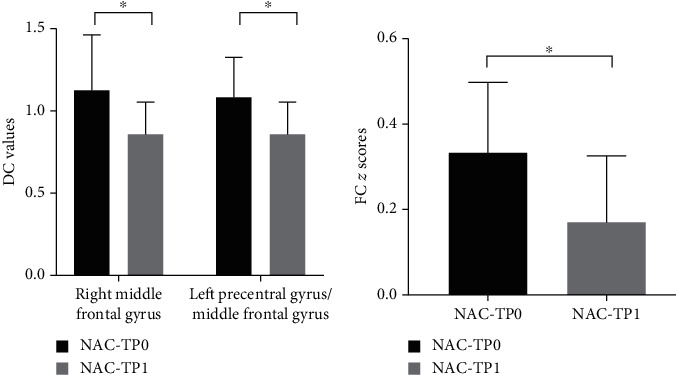
Comparisons of DC and FC between TP0 and TP1 in the NAC group (paired sample *t*-test). (a) Comparison of DC in the right middle frontal gyrus and left precentral gyrus/middle frontal gyrus between the TP0 and TP1 NAC groups. (b) Ordinates represent FC *z* scores between the left precentral gyrus/middle frontal gyrus and bilateral precuneus. ^∗^*P* < 0.05 (corrected with Gaussian random-field theory). Error bars define the SD.

There was no significant intragroup statistical difference in the DC values of the HC group and the NAC group at the two time points. In the NAC group, the DC values of the patients' right middle frontal gyrus and left precentral gyrus/middle frontal gyrus decreased significantly over time from TP0 to TP1 (Figure 2, 3(a) and Table 5). We selected the above regions as the seed regions and compared the gray matter volumes of seed regions in the NAC group. The results showed that the gray matter volumes of the right middle frontal gyrus and left precentral/middle frontal gyrus were 0.39 ± 0.06 cm^3^ and 0.38 ± 0.06 cm^3^ at TP0 and 0.30 ± 0.05 cm^3^ and 0.29 ± 0.05 cm^3^ at TP1, respectively. The gray matter volume of these two regions was significantly different at both times (*t* = 2.913, *P* = 0.006; *t* = 2.513, *P* = 0.017). Afterwards, the FC between the seed region and the whole brain voxel was analyzed. The results showed that FC between the left precentral gyrus/middle frontal gyrus and bilateral precuneus was significantly reduced from TP0 to TP1 (Figures 2 and 3(b) and Table 6). No brain regions with altered connectivity were detected in the right middle frontal gyrus in the NAC at TP1.

### 3.4. ROC Curve

**Figure 4:**
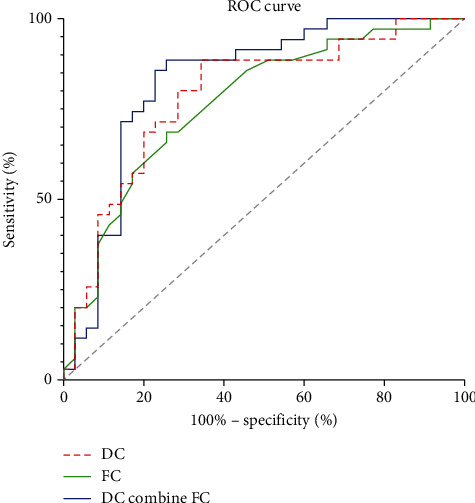
ROC curve analysis of the DC values and FC *z* scores for altered brain regions. The AUC of DC values in altered brain regions to distinguish between TP0 and TP1 was 0.7886 (*P* < 0.001; 95% CI: 0.6794-0.8978). The AUC of mean FC *z* scores to distinguish between TP0 and TP1 was 0.7665 (*P* < 0.001; 95% CI: 0.6546-0.8785). The AUC of combined DC values and FC *z* scores to distinguish between TP0 and TP1 was 0.8278 (*P* < 0.001; 95% CI: 0.6226-0.8607).

According to ROC curve analysis, the AUC of DC values and FC *z* scores to distinguish the status of breast cancer patients before and after chemotherapy (TP0 and TP1) were 0.7886 (*P* < 0.001; 95% CI: 0.6794-0.8978) and 0.7665 (*P* < 0.001; 95% CI: 0.6546 - 0.8785), respectively; the AUC of combined model of DC values and FC *z* scores was 0.8278. (*P* < 0.001; 95% CI: 0.6226-0.8607). (Figure 4).

### 3.5. Correlation Analysis

Previous results showed that the DC values and FC *z* scores of the right middle frontal gyrus and left precentral gyrus/middle frontal gyrus were significantly different between TP0 and TP1. We performed spearman correlation analysis between neuropsychological test scores (SAS) and average DC values of these two brain regions, as well as FC *z* scores. There was no significant correlation between them (*P* > 0.05).

## 4. Discussion

In the present study, DC and FC were used to assess intrinsic brain connectivity in breast cancer patients after the first cycle of NAC. These patients exhibited aberrant DC in the right middle frontal gyrus and left precentral gyrus/middle frontal gyrus. Taking the above brain regions as seed regions for whole-brain voxel-wise FC analysis, we found that FC between the left precentral/middle frontal gyrus and bilateral precuneus gyrus was reduced after the first cycle of NAC. Furthermore, the brain function indexes of altered brain region show satisfactory performance to distinguish between the patients before and after chemotherapy. These findings may help to improve the understanding of the mechanism of acute brain injury caused by NAC in breast cancer patients.

According to neuropsychological test, in the NAC group, we found that SAS was significantly reduced at TP1 compared to TP0, which was similar to the results of most previous studies [26–28]. Breast cancer patients who suffer more negative effects from the disease and treatment in the beginning experience a higher level of anxiety [29]. Over a period of time, breast cancer patients will gradually come to terms with the disease and the treatment procedures [30]. Moreover, a proportion of patients were relieved and showed lower anxiety levels due to the diminished lump size and reduced pain [31]. Even SAS scores were significantly different in TP0 and TP1 in the NAC group; the scores of all patients were less than 50, indicating that they had no anxiety experience and belonged to a normal state; it may be the underlying reason for the lack of anxiety-related changes in brain function. In addition, the DST forward scores of the NAC group at TP0 and TP1 were significantly lower than those of the HC group. The DST forwards is related to short-term memory. Previous studies have reported that cancer-related cognitive impairment includes short-term memory that occurs at various stages, including before, during, and after cancer treatment [32, 33]; this may be mediated by cancer-related posttraumatic stress [34].

Previous studies have proven that impaired executive function is one of the main manifestations of cognitive impairment in chemotherapy patients [3]. Although abnormalities of executive function were not reflected by neuropsychological test in our study, functional changes occurred in the brain areas related to executive function in patients. We observed a significant decrease in DC in the bilateral middle frontal gyri, which have been demonstrated to be critical to executive function [35]. We speculated that the brain may maintain relatively normal executive function through the compensatory effect in a short time after NAC. It is also possible that the scale is not sensitive to subtle differences in cognitive function [36]. Generally, the present study indicated that MRI can detect brain damage caused by chemotherapy earlier than scales, which is consistent with previous studies [37–39].

We also found reduced DC in the left precentral gyrus/middle frontal gyrus after the first cycle of NAC. The left precentral gyrus is one of the main motor areas in the cerebral cortex, and it is associated with other motor areas, such as the middle frontal gyrus, to plan and perform actions [40]. Previous studies on schizophrenia suggested that the precentral gyrus was associated with motor-related cognitive function [41]. The decreased DC of the left precentral gyrus/middle frontal gyrus after short-term NAC suggested that motor-related cognitive function may be vulnerable to chemotherapy attack.

To better understand the neural mechanisms underlying chemotherapy-induced brain damage, it is essential to consider how the brain regions work together rather than studying them in isolation [42]. The results showed that the DC distribution changed significantly in the brain area, and their gray matter volume also decreased significantly. Therefore, it is reliable to use these two brain regions as seed regions for whole-brain functional connectivity analysis. After the first cycle of NAC, we found that FC between the left precentral gyrus/middle frontal gyrus and bilateral precuneus was decreased in breast cancer patients. The precuneus is a critical node in the default mode network (DMN) [43]. Our results suggest that the balance between the DMN network and motor (precentral gyrus) and execution-related (middle frontal gyrus) brain regions may be disrupted, indicating that chemotherapy may reduce the ability to prepare for future task execution in patients with breast cancer. According to ROC curve results, brain function parameter DC and FC values can reflect the brain injury caused by chemotherapy and may be potential neuroimaging markers. However, this needs to be confirmed in subsequent longitudinal studies.

In our study, we observed significant changes in DC distribution in the NAC group in longitudinal comparison (TP0 vs. TP1) and nonsignificant differences in cross-sectional comparison (TP0 vs. HC; TP1 vs. HC). This may be due to the presence of confounding variables. Though there were no differences in important confounding variables (such as age, education, BMI, blood pressure, and menstrual status) between the HC and NAC groups at baseline, there may still exist other uncontrolled confounding variables. In addition, these findings might be underpowered to detect reliable cross-sectional associations owing to the small sample size. And longitudinal studies require far fewer participants than do cross-sectional studies to detect small changes in the brain [44, 45].

There are several limitations of this study. First, patients with breast cancer in this study received a variety of chemotherapy regimens, and they have different menstrual statuses. In addition, there was no significant correlation between neuropsychological test scores and brain function indicators, which may also be attributed to the above factors. The research cohort needs to be further expanded in future studies and grouped for further exploration. Second, the HC group did not receive scan at TP1, since the interval between the two scans was relatively short. Previous studies suggested that the brain function of HC did not change in a short time [16]. Therefore, we only scanned the HC group at TP0 and assumed that the brain function did not change with time. However, in future longitudinal studies, this should be treated more cautiously.

## 5. Conclusion

In the present study, DC and FC were used to characterize the pattern of brain dysfunction in breast cancer patients after the first cycle of NAC. We found that breast cancer patients exhibited aberrant brain connection in the right middle frontal gyrus and left precentral gyrus/middle frontal gyrus, which may be involved in executive function and motor-related cognitive function. Our results support the notion that changes in brain functional connectivity may precede changes in cognitive performance. However, these findings need to be further validated with larger cohorts and longer follow-up in future studies.

## Figures and Tables

**Table 1:** Clinical characteristics.

Characteristics	NAC group	HC group	*P* value
	(*n* = 35)	(*n* = 30)	
Age (years)	50.11 ± 9.19	50.77 ± 9.55	0.78
Education (years)	9.14 ± 3.75	10.03 ± 3.19	0.31
BMI (TP0)	24.05 ± 2.87	32.28 ± 44.03	0.32
Hypertension/nonhypertension (TP0)	6/29	3/27	0.49
Premenopausal/postmenopausal (TP0)	14/21	14/16	0.59
Stage			
cIIA/cIIB	2/4	N/A	N/A
cIIIA/cIIIb/cIIIc	17/5/7	N/A	N/A
Regimen			
AC-T	9	N/A	N/A
EC-T	3	N/A	N/A
TCbHP	7	N/A	N/A
TCbHB	4	N/A	N/A
TAC	12	N/A	N/A

Distribution data are reported as means ± SD. For the intergroup comparisons of age, education, and BMI, the *P* value was obtained using the independent sample *t*-test. For the intergroup comparisons of blood pressure and menstrual status, the *P* value was obtained using the *χ*^2^ test. Abbreviations: BMI: body mass index; AC-T: epirubicin, cyclophosphamide, and paclitaxel; EC-T: doxorubicin, cyclophosphamide, and paclitaxel; TCbHP: paclitaxel, carboplatin, pertuzumab, and trastuzumab; TCbHB: paclitaxel, carboplatin, trastuzumab, and pyrotinib; TAC: docetaxel, doxorubicin, and cyclophosphamide; N/A: not applicable.

**Table 2:** Comparison of the neuropsychological test results between the NAC group at TP0 and the HC group.

Neuropsychological test	NAC TP0	HC group	*P* value
	(*n* = 32)	(*n* = 30)	
General cognition			
FACT-Cog	113.05 (101.65, 118.60)	110.45 (94.95, 117.10)	0.28
PCI	60.30 (57.83, 64.80)	59.40 (51.30, 63.23)	0.12
Oth	16.00 (15.00, 16.00)	16.00 (14.00, 16.00)	0.42
PCA	21.00 (16.30, 21.80)	20.61 (16.13, 21.80)	0.96
QOL	16.00 (12.25, 16.00)	16.00 (15.00, 16.00)	0.28
Executive function and psychomotor speed			
TMT-A	56.50 (34.25, 90.00)	42.00 (30.00, 57.75)	0.11
Mental flexibility			
VFT	37.00 (31.00, 42.75)	32.50 (27.00, 39.25)	0.10
Working memory			
DST forwards	7.00 (6.00, 8.00)	10.00 (8.75, 12.00)	<0.001
DST backwards	4.00 (4.00, 5.00)	4.00 (4.00, 6.00)	0.63
Anxiety and depression			
SAS	27.50 (26.00, 32.00)	27.50 (25.00, 31.25)	0.79
SDS	26.00 (25.00, 30.00)	26.00 (25.00, 30.00)	0.94

Nonnormal distribution data are reported as [*M*(QR)]. For the intergroup comparison, the *P* value was obtained using the Mann–Whitney *U* test. Abbreviations: FACT-Cog: Functional Assessment of Cancer Therapy-Cognitive Function; PCI: perceived cognitive impairments; Oth: comments from others; PCA: perceived cognitive abilities; QOL: impact on quality of life; TMT-A: Trail Making Test A; VFT: Verbal Fluency Test; DST: Digital Span Test; SAS: Self-Rating Anxiety Scale; SDS: Self-Rating Depression Scale.

**Table 3:** Comparison of the neuropsychological test results between the NAC group at TP1 and the HC group.

Neuropsychological tests	NAC TP1	HC group	*P* value
	(*n* = 32)	(*n* = 30)	
General cognition			
FACT-Cog	110.40 (105.05, 115.80)	110.45 (94.95, 117.10)	0.99
PCI	62.55 (58.50, 63.90)	59.40 (51.30, 63.23)	0.12
Oth	16.00 (16.60, 16.00)	16.00 (14.00, 16.00)	0.05
PCA	19.05 (16.30, 21.00)	20.61 (16.13, 21.80)	0.33
QOL	15.00 (14.00, 16.00)	16.00 (15.00, 16.00)	0.13
Executive function and psychomotor speed			
TMT-A	46.50 (30.50, 71.50)	42.00 (30.00, 57.75)	0.50
Mental flexibility			
VFT	38.50 (28.50, 45.75)	32.50 (27.00, 39.25)	0.14
Working memory			
DST forwards	7.00 (6.00, 8.00)	10.00 (8.75, 12.00)	<0.001
DST backwards	4.00 (4.00, 5.00)	4.00 (4.00, 6.00)	0.88
Anxiety and depression			
SAS	26.00 (25.00, 28.00)	27.50 (25.00, 31.25)	0.20
SDS	27.00 (25.00, 31.00)	26.00 (25.00, 30.00)	0.83

Nonnormally distributed data are reported as [*M*(QR)]. For the intergroup comparison, the *P* value was obtained using the Mann–Whitney *U* test.

**Table 4:** Comparison of the neuropsychological test results between the TP0 and TP1 in the NAC group.

Neuropsychological tests	NAC TP0	NAC TP1	*P* value
	(*n* = 29)	(*n* = 29)	
General cognition			
FACT-Cog	112.50 (102.30, 118.60)	109.50 (104.55, 115.80)	0.60
PCI	60.30 (57.15, 64.80)	62.10 (58.05, 63.90)	0.95
Oth	16.00 (15.00, 16.00)	16.00 (16.00, 16.00)	0.15
PCA	16.00 (12.50, 16.00)	15.00 (14.00, 16.00)	0.37
QOL	21.00 (16.30, 21.80)	19.40 (16.30, 21.40)	0.57
Executive function and psychomotor speed			
TMT-A	60.00 (31.00, 90.00)	48.00 (30.00, 72.50)	0.24
Mental flexibility			
VFT	36.00 (30.50, 40.50)	39.00 (27.00, 46.00)	0.11
Working memory			
DST forwards	7.00 (6.00, 8.00)	7.00 (6.00, 8.00)	0.43
DST backwards	4.00 (4.00, 5.00)	4.00 (4.00, 5.00)	0.50
Anxiety and depression			
SAS	30.00 (26.00, 32.00)	26.00 (25.00, 27.50)	0.003
SDS	26.00 (25.00, 31.00)	27.00 (25.00, 31.00)	0.56

Nonnormal distribution data are reported as [*M*(QR)]. For the intragroup comparison, the *P* value was obtained using the Wilcoxon signed-rank test.

**Table 5:** Brain regions with significant DC differences between TP0 and TP1 in the NAC group.

Brain regions	BA	Peak MNI coordinates			*t*-value	Cluster (voxels)
		*x*	*y*	z		
Right middle frontal gyrus	46	42	39	33	-4.0392	56
Left precentral gyrus/middle frontal gyrus	6	-45	3	54	-4.285	100

MNI: Montreal Neurological Institute; BA: Brodmann area; *P* values were corrected with Gaussian random-field theory.

**Table 6:** Brain regions showing significant functional connectivity differences within the seed between TP0 and TP1 in the NAC group.

Seed regions	Brain regions	BA	Peak MNI coordinates			*t*-value	Cluster (voxels)
			*x*	*y*	*z*		
Right middle frontal gyrus	None						
Left precentral gyrus/middle frontal gyrus	Bilateral precuneus	Not applicable	-12	-63	30	-4.649	137

MNI: Montreal Neurological Institute; BA: Brodmann area; *P* values were corrected with Gaussian random-field theory.

## Data Availability

The study data may be available from Jiuquan Zhang (zhangjq_radiol@foxmail.com) upon reasonable request.

## Abbreviations

AUC: Area under the curve
BMI: Body mass index
DC: Degree centrality
DST: Digit Span Test
DMN: Default mode network
FACT-Cog: Functional Assessment of Cancer Therapy-Cognitive Function
FC: Functional connectivity
HC: Healthy control
NAC: Neoadjuvant chemotherapy
Oth: Comments from others
PCA: Perceived cognitive abilities
PCI: Perceived cognitive impairments
QOL: Impact on quality of life
ROC: Receiver operating characteristic
Rs-fMRI: Resting-state functional magnetic resonance imaging
SAS: Self-Rating Anxiety Scale
SDS: Self-Rating Depression Scale
TMT: Trail Making Test
TP: Time point
VFT: Verbal Fluency Test.
